# Relationships of Physical Activity, Depression, and Sleep with Cognitive Function in Community-Dwelling Older Adults

**DOI:** 10.3390/ijerph192315655

**Published:** 2022-11-25

**Authors:** Kahee Kim, Gyubeom Hwang, Yong Hyuk Cho, Eun Jwoo Kim, Ji Won Woang, Chang Hyung Hong, Sang Joon Son, Hyun Woong Roh

**Affiliations:** 1 Department of Psychiatry, Ajou University School of Medicine, Suwon 16499, Republic of Korea; 2 Suwon Geriatric Mental Health Center, Suwon 16499, Republic of Korea

**Keywords:** physical activity, depression, sleep, cognitive function, community-dwelling older adult

## Abstract

This cross-sectional, observational study aimed to integrate the analyses of relationships of physical activity, depression, and sleep with cognitive function in community-dwelling older adults using a single model. To this end, physical activity, sleep, depression, and cognitive function in 864 community-dwelling older adults from the Suwon Geriatric Mental Health Center were assessed using the International Physical Activity Questionnaire, Montgomery-Asberg Depression Rating Scale, Pittsburgh Sleep Quality Index, and Mini-Mental State Examination for Dementia Screening, respectively. Their sociodemographic characteristics were also recorded. After adjusting for confounders, multiple linear regression analysis was performed to investigate the effects of physical activity, sleep, and depression on cognitive function. Models 4, 5, 7, and 14 of PROCESS were applied to verify the mediating and moderating effects of all variables. Physical activity had a direct effect on cognitive function (effect = 0.97, *p* < 0.01) and indirect effect (effect = 0.36; confidence interval: 0.18, 0.57) through depression. Moreover, mediated moderation effects of sleep were confirmed in the pathways where physical activity affects cognitive function through depression (F-coeff = 13.37, *p* < 0.001). Furthermore, these relationships differed with age. Thus, the associations among physical activity, depression, and sleep are important in interventions for the cognitive function of community-dwelling older adults. Such interventions should focus on different factors depending on age.

## 1. Introduction

With the increasing life expectancy in developed countries, cognitive impairment is emerging as a major public health problem among older adults [1]. Cognitive impairment is associated with difficulties in performing daily activities and reduced quality of life [1]. Thus, the determination of modifiable factors to prevent cognitive decline and thereby ensure healthy living and active aging for older adults may be clinically important. In a meta-analysis of modifiable factors related to cognitive function and dementia [2], physical activity was considered an important modifiable factor [3,4,5]. Therefore, many studies have evaluated the effects of physical activity on cognitive function in older adults [6,7,8,9,10].

Several studies have been reported suggesting that physical activity, depression, sleep, cognitive function and prefrontal cortex (PFC) activity are related [11,12,13]. Physical activity can affect PFC activity, which can affect cognitive function [14]. PFC also appears to be linked to depression and sleep [15,16]. The fact that one of the frequently affected areas in major depressive disorders is the PFC suggests that PFC is also closely related to depression [17]. Furthermore, studies have reported that sleep deprivation reduces the functional connectivity of the PFC region [18]. The PFC region is widely interconnected with the hippocampus and amygdala of the brain, participating in higher-order decision making, planning and organizing the future, and playing an important role in cognitive function [19]. In addition, attempts are being made to explain these relationships by focusing on changes in autonomic regulation, cortical state, or brain oxygenation [20,21,22]. Taken together, it can be predicted that exercise, depression, sleep, and cognitive function can be closely influenced.

Cognitive decline has also been recently reported to be associated with depression, suggesting that cognitive impairment is an important aspect of depression [23,24]. In October 2017, the WHO announced that unipolar depression occurs in 7% of the general older adults population and accounts for 5.7% of adults who are over the age of 60. In particular, depression is a representative disease of old age that occurs in 20–30% of normal older adults [25]. Cognitive decline has been observed in older adults with depression, and several studies have suggested a close relationship between depression and cognitive function in older adults [26,27].

Various types of sleep disorders have also been reported to be associated with cognitive decline [28,29]. In particular, sleep fragmentation is associated with an annual cognitive decline of 22%, and an extremely small amount of sleep is also a risk for cognitive impairment in older adults [30]. Accumulating evidence indicates a strong association between sleep disturbance and cognitive impairment in older adults [31,32,33]. A relationship between depression and sleep has also been reported in several studies [34,35].

Many existing studies have reported the individual relationships of physical activity, depression, and sleep with cognitive function. However, few studies have simultaneously considered all three factors in terms of their relationships with cognitive function. Therefore, this study aimed to evaluate the relationship between exercise, depression, sleep, and cognitive function using a statistical model, thereby integrating our understanding of the relationship between these four variables.

## 2. Materials and Methods

### 2.1. Participants

This is a cross-sectional, observational study. The study sample consisted of 864 community-dwelling older persons, aged 60–90 years, who visited the Suwon Geriatric Mental Health Center in the Republic of Korea for a general assessment of cognitive impairment, depression, and other potential mental health issues. The subject collection process is shown in the Supplementary Figure S1. This study was conducted from January 2017 to April 2021. The exclusion criteria were as follows: a history of another psychiatric disorder (schizophrenia, bipolar disorder, or alcohol use disorder); severe cognitive impairment with a global deterioration score of >3 points or a Mini-Mental Status Examination score of <18 points; neurological or medical disorders, such as traumatic brain injury and brain tumor; difficulty in performing physical activities due to medical problems such as asthma, chronic obstructive pulmonary disease, complications of diabetes, or hemiparalysis; and missing values for independent, dependent, and covariates. The Institutional Review Board of Ajou University Hospital approved this study (AJIRB-SBR-SUR-16-122). Written informed consent was obtained from all of the participants.

### 2.2. Assessments and Measurements

#### 2.2.1. Physical Activity

In this study, the International Physical Activity Questionnaire (IPAQ) short form was used, and the participants were asked questions regarding three distinct activity types: walking, moderate-intensity activities, and vigorous-intensity activities. The questions were set up such that the scores for walking, moderate-intensity activity, and vigorous-intensity activity could be assigned separately. The total amount of activity during the week was calculated using the IPAQ scoring protocol [36]. IPAQ has been reported as a valid test method [37].

#### 2.2.2. Depressive Symptoms

The severity of depressive symptoms was assessed using the 10-item Montgomery-Asberg Depression Rating Scale (MADRS) (Montgomery and Asberg, 1979). Each item is scored by doctors on a 7-point scale (range 0–6), yielding a final score between 0 and 60. Higher scores indicate more serious disease. The validity of the K-MADRS was found to be satisfactory (Cronbach’s alpha = 0.79) [38].

#### 2.2.3. Sleep

The Korean variant of the Pittsburgh Sleep Quality Index (PSQI-K) was used to study insomnia. The PSQI-K is a popular and reliable tool for assessing sleep quality and consists of a self-report questionnaire on sleep quality over the previous month (Cronbach’s alpha coefficient = 0.69) [39]. Each of the seven subscores of the PSQI has a maximum score of 3 points [40]. The total score can range from 0 to 21, with 8 regarded as the passing mark [40]. 

#### 2.2.4. Cognitive Function

Cognitive function was evaluated using the Korean version of the Mini-Mental State Examination for Dementia Screening (MMSE-DS). The MMSE-DS survey items consisted of thirty questions in eight domains: time orientation, five questions; place orientation, five questions; memory, six questions; attention concentration, five questions; language ability, three questions; executive ability, three questions; spatiotemporal imagination, one question; and judgment and abstraction, two questions. The correct answer to each question was assigned one point for a total of thirty points, with lower scores indicating worse cognitive function [41]. MMSE-DS has been reported as a valid test method (Cronbach’s coefficient alpha = 0.826) [41].

### 2.3. Covariates

Age, sex, education, and living situation were all reported sociodemographic factors. Numerous studies have identified a correlation between physical disease and depression. Therefore, participants were questioned about their history of physical ailments such as hypertension, diabetes, and cardiovascular disorders (e.g., hyperlipidemia, heart failure, angina, myocardial infarction, and stroke).

### 2.4. Statistical Analysis

Numerical variables are presented as means and standard deviations and categorical variables as percentages and numbers. To investigate the associations of these factors with cognition, a multiple linear regression analysis was performed. The plug-in program PROCESS, created by Hayes (2013), employs an approach based on an ordinary least squares regression model and the bootstrap method and was used to investigate the statistical significance of the effects of mediation and moderation. Model 4 was used to simultaneously analyze the relationships of physical activity and depression with cognitive function, and models 5, 7, and 14 were used to verify the moderating mediation effect of sleep. A total of 10,000 bootstrap samples were used to test the statistical significance of the mediating variables. Using this methodology, an estimate of the indirect effect was produced. The significance threshold for this study was set at 0.05. The research data were analyzed using IBM SPSS v23.0 (SPSS Inc., Chicago, IL, USA).

## 3. Results

Table 1 presents the participants’ demographic characteristics. Among the 864 participants, 69.8% were female and 30.2% were male. Their mean age was 73.8 ± 6.9 years and mean years of education was 6.6 ± 4.6 years. Moreover, 40.6% of the participants lived alone, and 56.4%, 25%, and 39.7% had hypertension, diabetes, and cardiovascular disease as an underlying condition, respectively.

Table 2 presents the outcomes of multiple linear regression. Our multiple linear regression model showed an acceptable R square value and valid (R^2^ = 0.203, *p* = 0.001). Age, sex, education, living status, and the presence of physical illnesses such as hypertension, diabetes, and cardiovascular disease were the sociodemographic factors that were taken into consideration. Age and MADRS had a negatively significant relationship (estimate = −0.153, SE = 0.034, *p* = 0.000 and estimate = −0.183, SE = 0.036, *p* = 0.000, respectively), whilst education and IPAQ were positively significant (estimate = 0.335, SE = 0.034, *p* = 0.000 and estimate = 0.099, SE = 0.032, *p* = 0.002, respectively).

**Figure 1:**
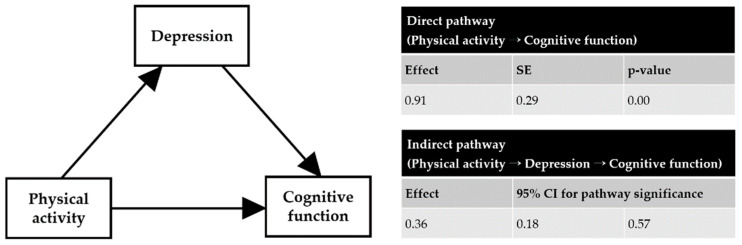
Effect of physical activity on cognitive function and the mediating effect of depression. Exercise had a direct effect on cognitive function. Depression had a partial mediated effect in this process. SE = standard error, CI = confidential interval.

Based on the findings of many studies, we assumed that physical activity would directly affect cognitive function, and we verified whether the mediating effect of depression was significant in this process (Figure 1). The results confirmed that physical activity had a direct positive effect on cognitive function (effect = 0.91, SE = 0.29, *p* < 0.01) (Figure 1). In addition, verification of the indirect pathway (physical activity → depression → cognitive function) confirmed that depression mediated the relationship between physical activity and cognitive function (Figure 1).

**Figure 2:**
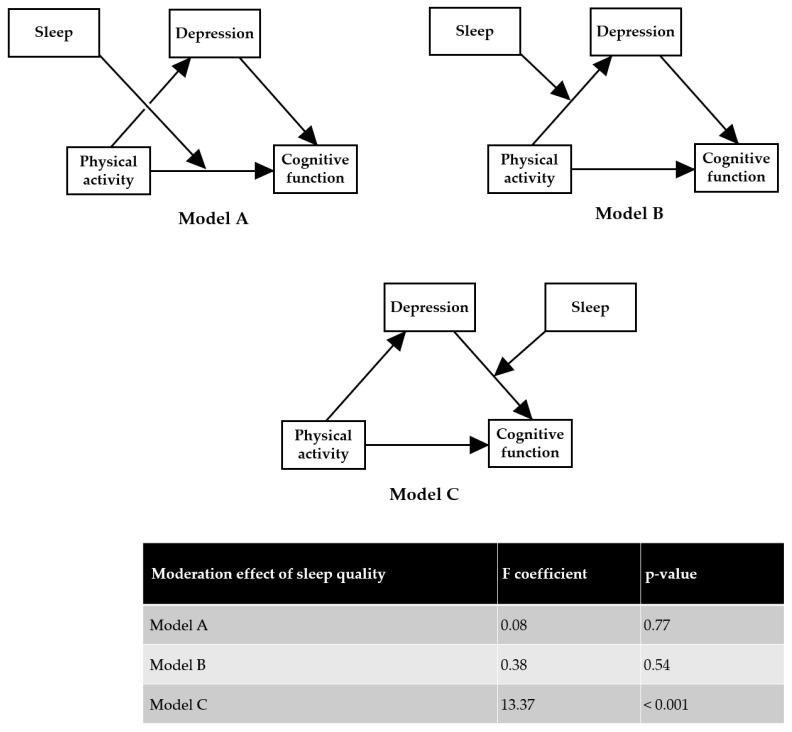
Moderating effect of sleep on the relationship of physical activity and depression with cognitive function. The mediated moderation effect of sleep was effective only between depression and cognitive function.

As confirmed in Figure 1, physical activity seems to directly affect cognitive function (physical activity → cognitive function) and indirectly (physical activity → depression → cognitive function). It was assumed that sleep would exhibit a moderating effect in the relationship between exercise, depression and cognitive function. Moreover, models A, B and C were set according to which stage sleep had a moderating effect (Figure 2). The results confirmed that only model C was significant (F-coeff = 13.37, *p* < 0.001) (Figure 2). Model A and B was not significant (F-coeff = 0.08, *p* = 0.77, and F-coeff = 0.38, *p* = 0.54, respectively).

**Figure 3:**
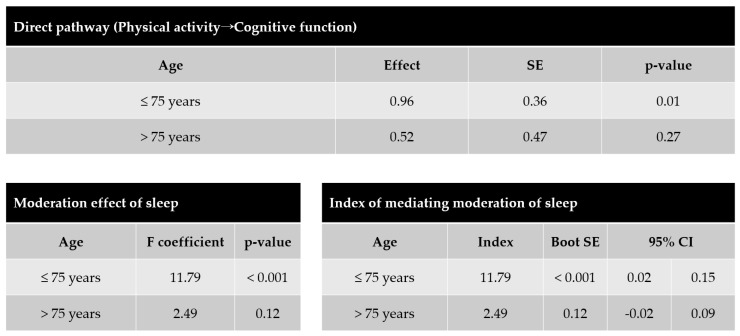
Analysis of the relationship changes in four factors with age. Model C of Figure 2 was significant under the age of 75, but not over the age of 75. SE, standard error; Boot SE, bootstrap standard error; CI, confidential interval.

Next, we verified whether the relationships among the four variables which were differed according to age (Figure 3). The direct effect of physical activity on cognitive function was significant in adults aged under 75 years (Effect = 0.96, SE = 0.36, *p* < 0.01), but not in those older than 75 years (Effect = 0.52, SE = 0.47, *p* > 0.05). For adults aged under 75 years, the moderating effect of sleep was significant (F-coeff = 11.79, *p* < 0.001), but it was not significant in older adults older than 75 years (F-coeff = 2.49, *p* = 0.12).

## 4. Discussion

In this study, we investigated the relationships of physical activity, depression and sleep in terms of their effects on cognitive function. Although many studies have reported the individual relationships of these factors with cognitive function, comprehensive simultaneous analyses of these relationships have been rarely performed. Therefore, we assessed the associations of physical activity, depression, and sleep with cognition simultaneously using one statistical model.

Several studies have reported that physical activity positively affects cognitive function. Similar results were observed in this study. We also found that physical activity in older adults had a significant effect on cognitive function by mediating depression. The positive effects of physical activity on depression appear to be mediated through psychosocial mechanisms such as social support and self-efficacy. Additionally, physical activity may be related to biological mechanisms that reduce inflammation, increase resilience to oxidative stress, or increase neuroplasticity, thereby showing positive effects on depression [42,43,44,45,46].

We hypothesized that sleep may play a moderating role in the effects of physical activity and depression on cognitive function and analyzed three models. The results showed the moderating effect of sleep between depression and cognitive function. In particular, the moderating effect of sleep was found only between depression and cognition. The indirect pathway was not significant in older adults with good sleep but was found to be significant in older adults with poor sleep.

The relationships of physical activity, depression, and sleep with cognitive function differed according to age. Physical activity had a direct effect on cognitive function in older adults aged under 75 years, but not in those older than 75 years. Considering the average physical activity amount of 786 for older adults older than 75 years and the average physical activity amount of 1013 for older adults aged under 75 years, older adults may find it difficult to perform a significant level of physical activity to improve cognitive function. In addition, sleep had a significant moderating mediation effect in adults aged under 75 years, but this effect was not significant in adults older than 75 years. This is thought to be the result of various factors intervening in cognitive function as well as greater vulnerability to various physical problems that can affect cognitive function, such as diabetes and vascular diseases.

The findings of this study confirmed that physical activity not only had a direct positive effect on cognitive function but also affected cognition through depression, and that it had a moderating effect on sleep in this process. In addition, this relationship appears to differ according to age. Thus, when evaluating various factors for improving cognitive function, the influence of age on different modifiable variables needs to be considered. For example, in older adults aged under 75 years, physical activity and sleep interventions can be expected to show preventive effects for cognitive decline. However, other interventions for depression may be more efficient in older adults older than 75 years.

Several possible mechanisms are conceivable for the relationship between physical activity, depression, sleep and cognitive function in our study. The first concern is the changes that occur as exercise affects the autonomic nervous system. Several studies have reported that physical activity has a therapeutic and protective effect on the autonomous system, and results have been suggested that physical activity can improve metabolic and autonomic function problems [47,48]. Furthermore, a study found that the autonomic nervous system dysfunction is associated with depression, and the level of the dysfunction is correlated with the severity of the depression [49]. Not only that, it also appears that impaired autonomic function is connected to decreased cognitive function [50,51,52]. These findings support the notion that exercise can affect depression and cognitive function by affecting the autonomic nervous system. Second, the effect of exercise on brain can be considered. Numerous studies have shown that physical activity alters brain plasticity and influences cognition [53]. Exercise has been shown to alter the shape of depressive patients’ brains, activate the activity of nearby brain regions, encourage behavioral adaptation, and preserve the volume of the hippocampal and white matter [44]. This enhances brain neuroprocessing and delays the onset of cognitive decline in depressive patients.

Further studies will be needed to confirm this mechanism. In addition to the evidence presented by us, there are attempts to explain changes in depression or cognitive function through changes in the metabolic system [54,55], and studies using EEG to evaluate brain plasticity and connectivity [56]. In addition, there are reports that the prefrontal Cortex and the autonomic nervous system mentioned above interact with each other [57]. These reports could provide multiple perspectives on the relationship between the four variables and provide the basis for further research using multiple techniques.

This study has several limitations. First, it was a cross-sectional study and showed limitations in proving the causal relationships among the four factors. Second, since this study was conducted with participants from a mental health center, the participants may have had a greater-than-normal incidence of psychiatric and physical symptoms, leading to a bias in the ability of this sample to represent the entire older adult population. Furthermore, we did not consider personality characteristics and anxiety that can affect the results due to limited sources. In addition, since we analyzed only the total amount of physical activity, we could not analyze the effects of intensity, type, and persistence of physical activity. Nevertheless, this study is meaningful in that it simultaneously analyzed the relationships of cognitive function with various modifiable factors that have been reported individually and summarized the relationships among the four factors. The findings suggest that interventions according to individual characteristics, such as age, may be important in improving the cognitive function of older adults in the community.

## 5. Conclusions

We simultaneously analyzed the relationships of physical activity, depression, and sleep with cognitive function, which have been evaluated individually in several studies. Physical activity had a direct positive effect on cognitive function and also affected cognitive function through depression. Additionally, the role of the moderating mediation effect of sleep in this process was confirmed. The relationships among the four variables seemed to change with age. Our study emphasizes the role of physical activity, depression, and sleep in interventions for improving the cognitive functions of older adults in the community and suggests that the focus on different factors may need to change depending on the age of the participants.

In this study, we attempted to analyze the four variables (activity, depression, sleep and cognitive function) considered in an integrated manner. However, in order for this study to have more significance, further evaluations of the mechanism that connects the four variables is needed. Many studies have been conducted to understand the relationship between each variable, and there are many studies to understand the mechanism. Such studies are paying attention to various perspectives such as brain plasticity, changes in cortical state, and autonomic function to explain the relationship between exercise, depression, sleep and cognitive function. The results of this study may help provide a framework for new ways to understand the relationship between psychophysiological factors for mental health of the older adults based on existing research.

## Figures and Tables

**Table 1:** Demographic characteristics of the study participants.

Variables ^a^	All Participants (*n* = 864)
Age, years	73.8 ± 6.9
Female (%)	603 (69.8)
Years of education	6.6 ± 4.6
Living alone (%)	351 (40.6)
Hypertension (%)	487 (56.4)
Diabetes (%)	216 (25.0)
Cardiovascular disease (%)	343 (39.7)
Korean Mini Nutritional Assessment score	25.2 ± 3.9
Korean Pittsburgh Sleep Quality Index	9.7 ± 4.2
Montgomery-Asberg Depression Rating Scale score	17.5 ± 10.7
International Physical Activity Questionnaire) ^b^ score	2.8 ± 0.4

^a^ Values represented as mean ± SD and number (%) for categorical variables. ^b^ Log transformation was performed due to skewness. SD = standard deviation.

**Table 2:** Multiple linear regression analysis for associations of cognitive function with sleep, physical activity, and depression.

	Dependent Variable: MMSE				
Independent Variables	Unstandardized Coefficient		Standardized Coefficient		
	β	Std. Error	β	Std. Error	*p*-Value
Age (years)	−0.086	0.019	−0.153	0.034	0.000
Sex	−0.129	0.278	−0.015	0.032	0.643
Education (years)	0.279	0.0.28	0.335	0.034	0.000
Living alone	−0.340	0.242	−0.043	0.031	0.160
Hypertension	0.106	0.249	0.014	0.033	0.672
Diabetes	−0.373	0.276	−0.042	0.031	0.177
Cardiovascular disease	0.062	0.247	0.008	0.032	0.802
PSQI	0.052	0.031	0.057	0.034	0.093
IPAQ	0.889	0.287	0.099	0.032	0.002
MADRS	−0.066	0.013	−0.183	0.036	0.000

PSQI = Pittsburgh Sleep Quality Index, IPAQ = International Physical Activity Questionnaire, MADRS = Montgomery-Asberg Depression Rating Scale, MMSE = Mini-Mental State Examination.

## Data Availability

The raw data supporting the conclusions of this article will be made available by the authors, without undue reservation.

## Supplementary Materials

The following supporting information can be downloaded at: https://www.mdpi.com/article/10.3390/ijerph192315655/s1, Figure S1: Flow chart of subjects after applying exclusion and inclusion criteria.
