# Supplementary material for: Relationships of Physical Activity, Depression, and Sleep with Cognitive Function in Community-Dwelling Older Adults
Source: Int J Environ Res Public Health. 2022 Nov 25;19(23):15655. doi: 10.3390/ijerph192315655 (PMC9737085; doi:10.3390/ijerph192315655)
Supplement: Supplementary file 1 [file ijerph-19-15655-s001.zip › ijerph-1929394-supplementary.pdf]

[Supplementary Figures and Tables]

Supplementary Figure S1. Flow chart of subjects after applying exclusion and inclusion criteria

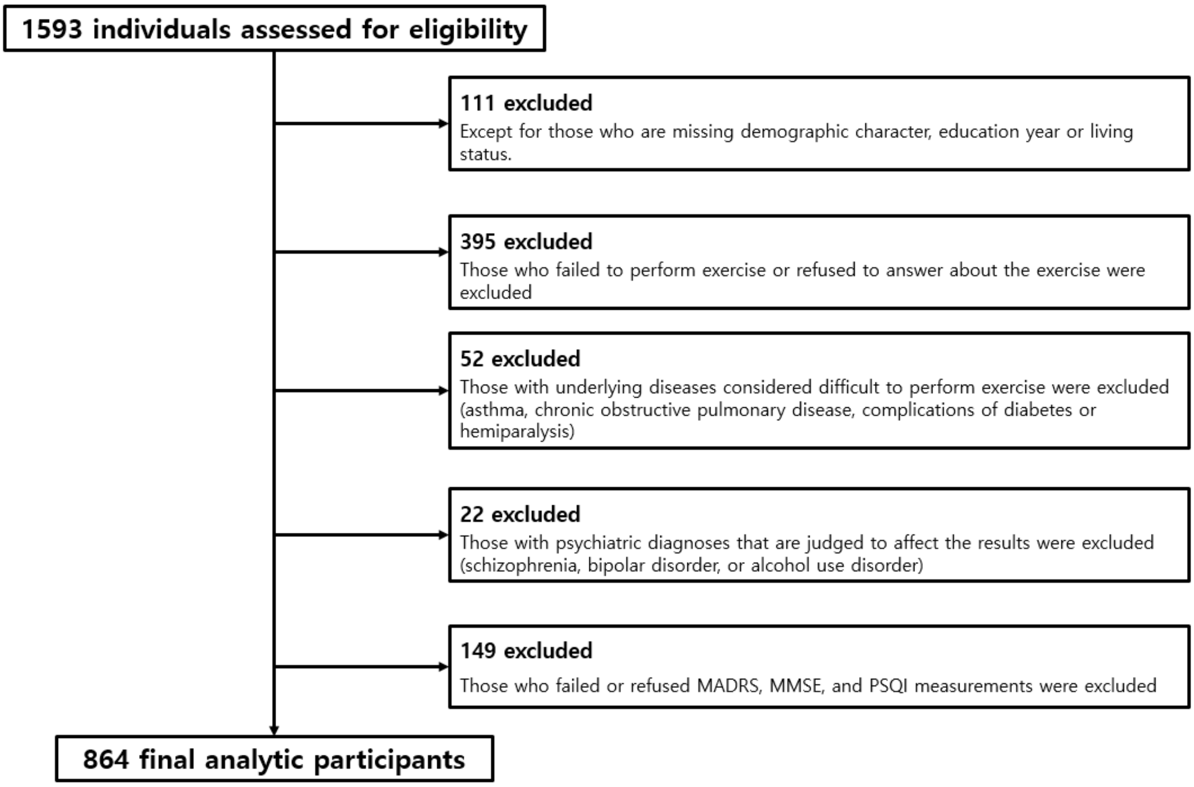

Abbreviations: PSQI = Pittsburgh Sleep Quality Index, MADRS = Montgomery-Asberg Depression Rating Scale, MMSE = Mini Mental State Examination
